# Subchondral Knee Injections: A Comprehensive Systematic Review of Current Clinical Outcomes, Safety, and Arthroplasty Delay

**DOI:** 10.1177/19476035261420389

**Published:** 2026-05-16

**Authors:** Yazan Abu Salem, Carlotta Franceschi, Pietro Conte, Giuseppe Anzillotti, Daniele D’Arrigo, Berardo Di Matteo, Elizaveta Kon

**Affiliations:** 1 IRCSS Humanitas Research Hospital, Rozzano, Italy; 2 Department of Biomedical Sciences, Humanitas University, Pieve Emanuele, Italy

**Keywords:** knee osteoarthritis, intraosseous injection, subchondral bone, bone marrow lesions, biologic therapy, platelet-rich plasma (PRP), mesenchymal stem cells (MSCs), bone marrow concentrate (BMC), subchondroplasty, calcium phosphate (CaP)

## Abstract

**Introduction:**

Osteoarthritis is considered a whole-joint disease involving subchondral bone. Intraosseous therapies such as calcium phosphate (CaP), platelet-rich plasma (PRP), and mesenchymal stem cells (MSCs) offer joint-preserving options for knee osteoarthritis (OA).

**Purpose:**

To critically appraise and update the clinical evidence on intraosseous injections for knee osteoarthritis, focusing on safety, functional outcomes, need for retreatment, and conversion rates to total knee arthroplasty (TKA).

**Methods:**

A systematic review of PubMed, Embase, and Cochrane was conducted following PRISMA guidelines and PROSPERO registration (CRD420251104989). Clinical studies reporting outcomes of intraosseous injections with CaP, PRP, or MSCs for knee osteoarthritis with ≥5 patients and ≥6 months of follow-up were included. Methodological quality was assessed using the modified Coleman Methodology Score and Cochrane Risk of Bias 2.0 tool.

**Results:**

Twenty-four studies involving 1,109 patients (mean age, 55 years; mean follow-up, 38 months) met inclusion criteria: 10 on CaP, 6 on PRP, and 8 on MSCs. Five were randomized controlled trials (RCTs). Most studies reported significant improvements in pain and function. CaP injection outcomes were variable, with TKA conversion rates ranging from 1.3% to 45%. PRP and MSCs studies showed favorable safety profiles and lower conversion rates. Long-term MSCs data indicated sustained relief and delayed TKA over up to 15 years. However, overall study quality was modest, with only one RCT rated as low risk of bias.

**Conclusion:**

Intraosseous injections may improve symptoms and delay arthroplasty in selected patients with knee OA, with MSCs showing the most favorable long-term results. PRP appears to be a safe option whereas CaP outcomes are more variable. Standardized protocols and high-quality RCTs with long-term follow-up are needed to optimize patient selection and treatment efficacy.

## Introduction

Osteoarthritis (OA) is a chronic joint disease marked by progressive degeneration of articular cartilage, often involving the subchondral bone and synovial structures. Its knee involvement represents a major cause of adult disability due to persistent pain, limited mobility, and impaired quality of life.^1-3^

Although total knee arthroplasty (TKA) remains the most effective treatment for end-stage disease, a significant proportion of patients, particularly those who are younger or more active, seek to delay or avoid surgery.^4,5^ Consequently, interest has grown in joint-preserving therapies that not only relieve symptoms but also potentially modify the course of the disease.^6-9^

Injectable therapies have become increasingly important in the management of early to moderate OA, with conventional options showing temporary benefits and growing interest in biologic-based treatments for their regenerative potential and minimally invasive profile^10-13^

Recent insights into OA pathophysiology have highlighted the central role of the osteochondral unit, particularly the subchondral bone, in both symptom generation and structural progression.^14^ Bone marrow lesions (BMLs), identifiable through a high signal intensity within the bone marrow region on fat-suppressed T2-weighted images and a low signal intensity on T1-weighted images, are radiological alterations often associated with the OA process, that have been proven to significantly affect pain perception, cartilage degeneration, and an increased risk of TKA.^15,16^ The subchondral rich innervation and vascularity point out toward a potential target for therapeutic intervention.^17^

Intraosseous injection techniques have emerged as a minimally invasive means of managing persistent BMLs and enhancing the condition of subchondral bone without significantly disrupting its natural structure. These injections are usually fluoroscopy-guided to accurately place the needle and ensure effective distribution of the therapeutic material within the targeted subchondral zone.^18-20^

Currently, few substances have been exploited for this therapeutical approach in the setting of OA-associated BMLs: calcium phosphate (CaP) intraosseous injections, which employs the material’s capabilities to support bone remodeling and reinforce subchondral integrity;^21,22^ Platelet-rich plasma (PRP), an autologous blood derivative rich in growth factors that stimulate the osteogenic activity of local mesenchymal stem cells (MSCs);^12,23,24^ MSCs, a cellular therapy obtained through bone marrow aspiration or from adipose tissue which has shown promise in enhancing bone repair and re-establishing subchondral balance.^25-27^

Despite growing interest, the current clinical evidence is fragmented and varies widely in terms of protocols, patient selection, and outcome measures. Moreover, the long-term safety and efficacy of intraosseous treatments, particularly their capacity to delay or prevent TKA, have not been systematically evaluated.^8,28,29^

Hence, the purpose of the present study is to systematically collect all the available evidence on knee intraosseous injections of CaP, PRP, and MSCs in patients with knee OA. Particular attention is given to clinical outcomes, complication rates, the need for retreatment, and conversion to TKA, with the goal of determining whether advances in technique or biologic strategies have enhanced the therapeutic potential of this minimally invasive approach.

## Material and Methods

This systematic review was registered with the International Prospective Register of Systematic Reviews PROSPERO 2025 CRD420251104989. A comprehensive search of 3 medical electronic databases (PubMed, Embase, and Cochrane) was carried out as part of this systematic review on February 12, 2025. To achieve the maximum search strategy sensitivity we combined keywords with Boolean operators “OR” or “AND” for the literature terms “calcium phosphate,” “tricalcium,” “bone substitute,” “bony substitute,” “PRP,” “ACP,” “platelet-rich,” “Accufill,” “platelet derived,” “platelet concentrate,” “growth factor,” “stem cells,” “mesenchymal,” “adipose,” “BMC,” “bone marrow concentrate,” “SVF,” with the terms “subchondroplasty,” “subchondral injection,” “subchondral,” “intraosseous,” “knee injection” and “knee intraosseous injection”. The complete search strategi is detailed in Appendix A.

A total of 19,571 potential articles were identified through our database search.

The following inclusion criteria were adopted for study selection: clinical trials of any level of evidence, written in English, and reporting clinical results following knee intraosseous injections of bone substitutes (CaP) or biologic agents (PRP or MSCs products), with a minimum number of 5 patients treated and a minimum follow-up period of 6 months. A 6-month follow-up was accepted as the final endpoint for study inclusion. When studies reported outcomes at multiple time points, the longest available follow-up was extracted and considered for analysis.

We excluded all articles coming from non-peer reviewed journals, surveys, editorials, special topics, conference presentations, narrative reviews, articles where the access to the full text was blocked and case reports or mini case series (<5 patients).

After title and abstract screening, 129 studies were assessed for eligibility. Full text was retrieved, and after a deep and careful review, 112 articles were excluded because they were not related to the knee joint, described only intra-articular procedures, presented less than 5 cases, or reported an insufficient period of follow-up.

**Figure 1.**
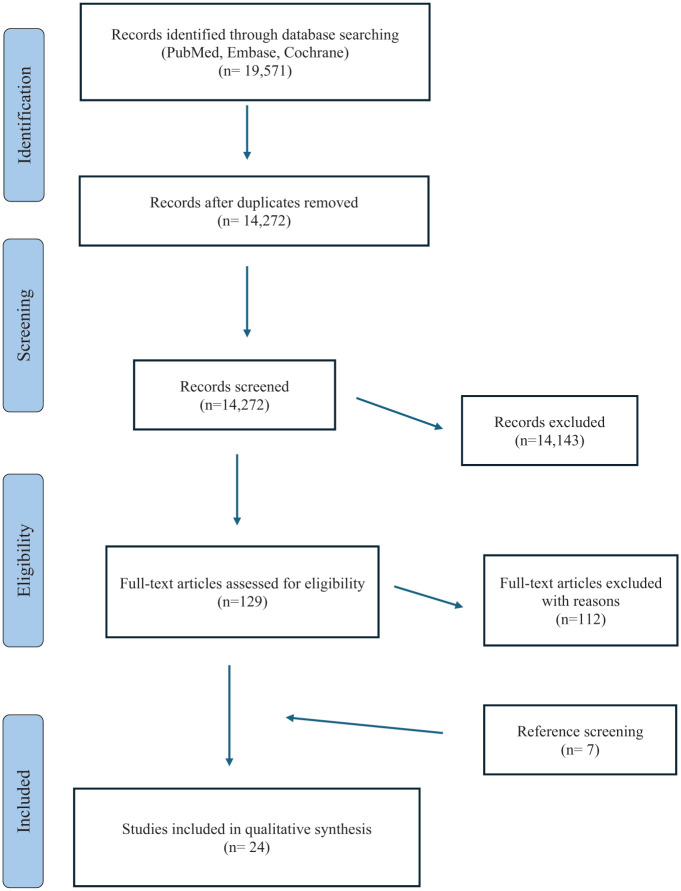
PRISMA.

All the references of the retrieved articles were further reviewed for identification of potentially relevant studies and reassessed using the same inclusion and exclusion criteria stated above: 7 additional studies were included from the references. A PRISMA (Preferred Reporting Items for Systematic Reviews and Meta-Analyses) flowchart of the selection and screening process is provided in **Figure 1**.

All data were extracted and reviewed from article texts, tables, and figures by 2 independent investigators (YAS and CF). Two additional reviewers (PC and GA) assisted in data verification and analysis. The results were reviewed by the senior investigators (BDM and EK).

Data regarding indication criteria for intraosseous injections were extracted when available, including the presence of MRI-detected bone marrow lesions (BMLs), lesion size, number, and anatomical location, as well as prior failure of non-operative treatments. However, these parameters were inconsistently reported across studies and lacked standardized definitions, precluding a formal quantitative or subgroup analysis based on lesion characteristics or treatment indications.

Risk of bias and quality assessment of the included articles was done following the Coleman Methodology Score modified by Kon *et al*.^30^ and the Cochrane Risk of Bias 2.0 tool (RoB 2) tool by Sterne *et al*.^31^ for the randomized controlled trials (RCTs). The assessment was independently performed by 2 authors (YAS and CF). Any divergence was discussed with the senior investigators, who made the final judgment.

## Results

According to the inclusion and exclusion criteria, we identified a total of 24 studies. Ten studies^32-41^ involved the use of bone substitutes (CaP), 8^42-49^ involved injection of MSCs products (in 2 studies^46,49^ there was the combination with PRP and 1 study^48^ a combination with core decompression and autologous bone graft) and 6 studies^23,50-54^ used the injection of PRP.

### Quality Assessment for Non-Randomized Studies

Study quality was assessed using the modified Coleman Methodology Score^30^ for the 19 non-randomized studies. Results of the quality assessment using the modified Coleman Methodology Score are detailed in **Table 1** for each study. The average score was 44/100, indicating overall poor methodological quality across the included studies.

**Table 1.** Quality Assessment Using the Modified Coleman Methodology Score by Kon *et al*.^30^

Study	Total Score	Study Size	Mean Follow-up	Other Procedures	Type of Study	Procedure Description	Postop Rehab	MRI Outcome	Histological Outcome	Outcome Criteria	Outcome Assessment	Selection Process
Bonadio *et al*.^32^	36	0	0	10	10	5	0	0	0	5	3	3
Byrd *et al*.^33^	21	10	5	0	0	0	0	0	0	0	3	3
Chua *et al*.^34^	38	0	2	10	10	5	0	0	0	5	3	3
Chatterjee *et al*.^35^	31	4	2	4	0	5	0	0	0	5	3	8
Cohen and Sharkey^36^	41	10	2	10	0	3	2	0	0	5	3	6
Davis *et al*.^37^	20	7	2	0	0	0	0	0	0	5	3	3
Farr and Cohen 2013^38^	34	7	2	0	10	5	2	0	0	5	3	0
Vad *et al*.^42^	48	0	2	10	10	5	0	10	0	5	3	3
Sánchez *et al*.^53^	48	4	2	10	10	5	0	0	0	5	9	3
Sánchez *et al*.^54^	47	0	0	10	10	5	0	0	0	5	9	8
Centeno *et al*.^49^	61	7	5	7	10	5	5	10	0	5	4	3
Pearl *et al*.^46^	38	4	5	10	0	5	0	0	0	5	3	6
Kon *et al*.^47^	58	4	5	7	10	5	5	5	0	5	4	8
Dallo *et al*.^48^	52	0	2	7	10	5	5	5	0	5	7	6
Araujo *et al*.^51^	31	4	2	7	0	5	0	0	0	5	2	6
Lychagin *et al*.^52^	57	0	2	10	10	5	0	10	0	5	7	8
Stratton *et al*.^39^	48	7	5	10	0	5	5	0	0	5	3	8
Randelli *et al*.^40^	62	7	5	7	10	5	5	5	0	5	7	6
Di Matteo *et al*.^41^	57	10	2	10	10	5	5	0	0	5	2	8

### Quality Assessment for Randomized Controlled Studies

For the 5 RCTs, quality assessment was performed using the Cochrane Risk of Bias 2.0 tool (RoB 2).^31^ The study by Su *et al*.^23^ was judged to have an overall *high risk of bias*, with *high risk* in deviations from intended interventions and outcome measurement, and *some concerns* regarding the randomization process and selective reporting. The RCT by Hernigou *et al*.^45^ was rated as having *some concerns* overall due to *some concerns* in outcome measurement and reporting, despite *low risk* in other domains. The remaining 3 RCTs, 2 by Hernigou *et al*,^43,44^ and one by Barman *et al*.^50^ were all assessed as *low risk* in the domains of randomization, intervention adherence, missing data, and selective reporting. However, 2 of these studies (Hernigou *et al*.^43^ and Barman *et al*.^50^) were judged to have *some concerns* in outcome measurement due to lack of assessor blinding, resulting in an overall judgment of *some concerns*, while Hernigou *et al*.^44^ was rated as *low risk of bias* overall. A detailed summary is presented in **Table 2.**

**Table 2.** Cochrane Risk of Bias 2.0 tool (RoB 2) by Sterne *et al*.^31^

Study (Author)	D1	D2	D3	D4	D5	Overall
Hernigou *et al*.^44^	●	●	●	●	●	●
Hernigou *et al*.^43^	●	●	●	●	●	●
Barman *et al*.^50^	●	●	●	●	●	●
Su *et al*.^23^	●	●	●	●	●	●
Hernigou *et al*.^45^	●	●	●	●	●	●

Legend

● Low risk.

● Some Concerns.

● High risk.

### Patients’ Demographics

The studies included a total of 1,109 patients, with a mean age of 55 years (range 28-75) and an average follow-up of 38 months (range 6–180 months) as shown in **Table 3**. Most of the studies had a mean follow-up around 14 months except for Bonadio *et al*,^32^ Barman *et al*.^50^ and Sánchez *et al*.^55^ who evaluated their patients up to 6 months, while Hernigou *et al*.^43-45^ in 3 studies evaluated their patients between 144 and 180 months. Eighteen out of 24 studies reported data on the grade of knee OA (Kellgren-Lawrence, Ahlback scale, YS scale) of the treated patients, with most of the cases classified as grade II or III (**Table 3**).

**Table 3.** Patients’ Demographics.

Study	Level of Evidence	Product	Other Procedures	Number of Cases	Median Age (Years)	Mean Follow-up (Months)	Body Mass Index (kg/m²)	OA Level
Bonadio *et al*.^32^	IV	CaP Graftys HBS	None	5	67.7	6	N/A	3 grade 1 KL, 2 grade 2 KL
Byrd *et al*.^33^	IV	CaP (Zimmer Knee Creations)	None	133	57	23.4 (range 14.6–32.1)	N/A	N/A
Chua *et al*.^34^	IV	CaP (Zimmer Knee Creations)	None	12	54	12	28.3	10 grade 2 KL, 2 grade 3 KL
Chatterjee *et al*.^35^	IV	CaP (Zimmer Knee Creations)	18/22 meniscectomy	22	53.5	12 (range 6–24)	29.7	2 grade 0 KL, 3 grade 1 KL, 9 grade 2 KL, 8 grade 3 KL
Cohen and Sharkey^36^	IV	CaP (Zimmer Knee Creations)	Arthroscopy	66	55.9	24	30.1	N/A
Davis *et al*.^37^	IV	CaP (Zimmer Knee Creations)	None	50	55	14.6 (range 12.9–25.1)	N/A	N/A
Farr and Cohen^38^	IV	CaP (Zimmer Knee Creations)	Arthroscopy	59	55.6	14.7	30.3	N/A
Stratton *et al*.^39^	IV	CaP	Arthroscopy (control)	53	60.25	24	29.9	Grade 0-3 KL
Randelli *et al*.^40^	IV	CaP	29/50 meniscectomy	50	66	26	26.11	7 grade 0, 16 grade 1, 20 grade 2, 7 grade 3
Di Matteo *et al*.^41^	II	CaP (Zimmer Knee Creations)	None	80	54	12	28 ± 4.6	27 grade 1, 32 grade 2, 21 grade 3 KL
Vad *et al*.^42^	IV	MSC PeCaBoo delivery system	None	10	63.5	14	N/A	N/A
Hernigou *et al*.^45^	II	MSC from BM	None	30	28	144	N/A	N/A
Hernigou *et al*.^43^	II	MSC from BM	TKA in controlateral knee (control)	140	75.4	180	28.1	36 grade 2 KL, 68 grade 3 KL, 36 grade 4 KL
Centeno *et al*.^49^	II	MSC from BM + PRP	IA	40	61	24	27.2-28.2	Grade 5-6 YS
Pearl *et al*.^46^	IV	MSC from BM + PRP	None	23	58	24	33.35	4 grade 3 KL, 19 grade 4 KL
Hernigou *et al*.^44^	II	MSC from BM	IA	120	61	180	28.1	22 KL grade 1, 40 KL grade 2, 38 KL grade 3, 20 KL grade 4
Kon *et al*.^48^	II	MSC from BM	IA	30	56.4	24	25.5	11 KL grade 2, 19 KL grade 3
Dallo *et al*.^49^	II	MSC from BM	Core decompression, autologous bone graft	15	54	12	25.17	Grade 2-3 KL
Barman *et al*.^50^	II	PRP	IA	50	57.6	6	25.53	50 grade 3 KL
Araujo *et al*.^51^	IV	PRP	IA	33	65.67	12.92	27.09	grade 2-3 Ahlback
Lychagin *et al*.^52^	III	PRP	None	17	41.7	12	<35	5 grade 2 KL, 10 grade 3 KL, 2 grade 4 KL
Su *et al*.^23^	II	PRP	IA	27	50.67	18	28	16 grade 2 KL, 11 grade 3 KL
Sánchez *et al*.^53^	III	PRP	IA	30	63.4	12	31	27 grade 3 AL, 3 grade 4 AL
Sánchez *et al*.^54^	IV	PRP	IA	14	62	6	20–33	9 grade 3 AL, 5 grade 4 AL

The indication for intraosseous treatment varied substantially among the included studies. Most investigations targeted patients with symptomatic knee OA and MRI-detected bone marrow lesions; however, the presence of BMLs was not uniformly required or consistently characterized. When described, lesions differed widely in size, number, and anatomical location within the femoral condyle or tibial plateau.

Detailed quantitative assessment of BML volume or extent was rarely performed, with standardized MRI-based grading systems reported infrequently. Similarly, prior failure of conservative treatment modalities, such as physical therapy, pharmacological management, or intra-articular injections, was inconsistently described. As a result, a precise definition of patient selection criteria for intraosseous injections could not be established from the available evidence.

Concerning the study design, 13 studies were prospective,^32,34,38,40-42,47-49,51-54^ 6 were retrospective,^33,35-37,39,46^ whereas the remaining 5 studies were randomized controlled trials (RCTs).^23,43-45,50^

### Bone Substitutes (CaP)

Seven studies^33-38,41^ (**Table 4**) investigated the outcome of CaP bone substitute Subchondroplasty® (SCP; Zimmer Knee Creations, USA), 1 study^32^ adopted the Graftys HBS (Graftys, France) and 2 studies^39,40^ did not provide details of the product.

**Table 4.** CaP Bone Substitute Subchondroplasty.

Study	Mean Follow-up (Months)	Results	Conversion to TKA	Complication	Need of Conservative Treatment
Bonadio *et al*.^32^	6	KOOS improved by 32.82 (*P* < 0.05), VAS improved by 7.2 (*P* < 0.05)	N/A	1/5 had cement extravasation with local pain for 1 week	None
Byrd *et al*.^33^	23.4 (range 14.6–32.1)	VAS improved by 4.9	25% (32/128)	N/A	35% short-term required unspecified injections; 41% mid-term
Chua *et al*.^34^	12	VAS improved by 5.4 (*P* < 0.001); KOOS improved by 34.7 (*P* < 0.001); WOMAC 47.8→14.3	N/A	1/12 cannula broke within bone during removal	N/A
Chatterjee *et al*.^35^	12 (range 6–24)	KOOS improved by 31.8; TLKSS improved by 29.5	45% (10/22)	None	8 received postoperative HA injections
Cohen and Sharkey^36^	24	VAS improved by 4.5; IKDC improved by 17.8; SF-12 improved by 6.9	30% (18/60)	1/60 drainage at injection site; 1/60 DVT	N/A
Davis *et al*.^37^	14.6 (range 12.9–25.1)	VAS improved by 4.7	8% (4/50)	2 repeated episodes of knee swelling	18/50 required HA + corticosteroid; 2 required serial aspiration
Farr and Cohen^38^	14.7	VAS improved by 4.4; IKDC improved by 22.4; SF-12 improved by 6.9 6 mo	25.4% (15/59)	None	N/A
Stratton *et al*.^39^	24	KOOS, JR improved by a 20.7 (*P* < 0.001)	11.3% (5/52)	N/A	N/A
Randelli *et al*.^40^	26	NRS improved by 3.95 at 6 mo; WOMAC by 28.2; IKDC by 30.7	22.9% (11/48)	5 intra-articular and 1 extra-articular leakage	7/48 patients required corticosteroid or hyaluronic injections
Di Matteo *et al*.^41^	12	KOOS Pain improved by 29.4; NRS by 2.7; KOOS-ADL by 26.9	1.3% (4/79)	9 minor adverse events (hemarthrosis, swelling, leakage, pain requiring medication or arthrocentesis)	1/79 patient had arthrocentesis and steroid injection

Five studies were prospective case series,^32,34,38,40,41^ whereas 5 had a retrospective design.^33,35-37,39^ Arthroscopic control to assess the eventual extravasation of CaP into the joint was performed in 5 cases,^34-36,38,40^ whereas Bonadio *et al*,^32^ Stratton *et al*.^39^ and Di Matteo *et al*.^41^ used just fluoroscopy to this purpose, and the remaining two^33,37^ articles did not provide details.

In 4 cases, cartilage and meniscus procedures were concurrently performed.^35,36,38,40^

### Clinical Findings

A statistically significant improvement in pain and function of the treated knees was a common finding among the included trials, as detailed in **Table 4**. Chatterjee *et al*.^35^ showed poor results, with 10 patients out of 22 (45%) requiring conversion to arthroplasty within 1 year. Cohen and Sharkey^36^ had a 30% conversion rate within 2 years.

Focusing on complications it was found that only 4% of patients experienced adverse events. In 2 cases, there was extra-articular cement extravasation, which did not require further surgical treatment and was managed just by analgesic drugs in the postoperative phases; Randelli *et al*.^40^ experienced in 5 cases intra-articular cement leakage. Cohen and Sharkey^36^ reported 1 case of postoperative drainage, which required surgical irrigation debridement, and 1 episode of deep venous thrombosis. Davis *et al*.^37^ reported 2 patients with repeated episodes of knee swelling, requiring multiple aspirations. Chua *et al*.^34^ reported during removal the breakage of the injective cannula within the bone due to excessive knee manipulation. Finally, Di Matteo *et al*.^41^ reported 9 minor adverse events: hemarthrosis, swelling, leakage, pain requiring medication or arthrocentesis.

With regard to the need of subsequent conservative retreatment, Byrd *et al*.^33^ reported that 41% of patients sought for further intra-articular injections in the following 3 years, Davis *et al*.^37^ reported 18 patients (36%) requiring hyaluronic acid (HA) or cortisone injection during the 24-month evaluation period, in Chatterjee *et al*,^35^ 8 patients required HA injection; Randelli *et al*.^40^ reported that 16% of patients required corticosteroid or hyaluronic injections and only 1 patient needed steroid injection in Di Matteo *et al*.^41^

As for TKA conversion rate, it ranged from 8% at a mean of 14 months’ follow-up^42^ to 45% at a mean of 12 months’ follow-up.^35^ Four studies^33,36,38^ showed a similar percentage of conversion to arthroplasty (22%–30%) at comparable time intervals (approximately 24 months after treatment). Stratton *et al*^39^ and Di Matteo *et al*.^41^ showed a conversion rate, respectively, of 11% at 24 months and 1.3% at 12 months’ follow-up. In 2 studies, no data on the TKA conversion rate were available.^32,34^

### Platelet-Rich Plasma

Six studies were included (Table 5) in this review that analyzed the results of knee intraosseous PRP injections.^23,50-54^ Sánchez *et al*. first performed a pilot trial^54^ and then a comparative study^53^ between 2 different treatment modalities: 1 subchondral injection of PRP followed by 2 intra-articular injections of the same PRP preparation 1 week apart versus 3 simple intra-articular injections. In the case of subchondral injections, 5 mL of PRP was applied both at the femoral and tibial bone-cartilage interface, while 8 mL of PRP was used for intra-articular delivery.

**Table 5.** PRP.

Study	Follow-up (Months)	Results	Conversion to TKA	Complications	Need for Conservative Treatment
Su *et al*.^23^	18	Significantly better results in intraosseous PRP group versus intra-articular PRP and HA	N/A	5 had knee pain and swelling	N/A
Sánchez *et al*.^53^	12	Intraosseous PRP superior to intra-articular PRP	16.7% required other interventions versus 26.7% in IA group	None	N/A
Sánchez *et al*.^54^	6	VAS +3.9, KOOS +13.05	14.2% (2/14)	1 had knee pain at 3 months	N/A
Araujo *et al*.^51^	12,9	WOMAC improved from 44.35 to 22.81; VAS from 5.79 to 2.41 (*P* < 0.0001)	N/A	N/A	N/A
Lychagin *et al*.^52^	12	VAS decreased from 51.4 to 11.1; WOMAC from 65.1 to 55.4; KOOS from 52.8 to 77.0	N/A	N/A	N/A
Barman *et al*.^50^	6	VAS improved from 6.4 to 3.6; KOOS P from 49.88 to 70.08	N/A	N/A	IO+IA-PRP consumed a greater number of Acetaphenomen.

Su *et al*.^23^ performed a 3-arm RCT comparing (a) intraosseous injections of 2 mL of PRP (2 administration at 14-day intervals), (b) intra-articular injections of 2 mL of PRP (2 administrations at 14-day intervals), and (c) intra-articular injections of HA (5 administration at 1-week intervals).

Lychagin *et al*.^52^ performed a prospective case–control study to assess the short-term effectiveness of intraosseous PRP injections, within the BML of individuals affected by OA.

Barman *et al*.^50^ in their RCT investigated whether adding IO-PRP injections to IA-PRP injections improves outcomes in patients with knee OA. A single-blind RCT where there were 2 groups: the IO + IA-PRP group that received 18 mL of PRP, 5 mL injected intra-osseously into the tibial plateau, 5 mL into the femoral condyle, and 8 mL intra-articularly; the IA-PRP group that received 8 mL of PRP intra-articularly only.

In their retrospective case series, Araujo *et al*.^51^ assessed the functional effects and pain relief in patients with grade II–III Kellgren-Lawrence (KL) knee OA treated with IO and IA injections of PRP, followed by 2 IA injections of HA at weeks 3 and 4.

### Clinical Findings

Both the comparative study by Sánchez *et al*.^53,54^ and the RCT by Su *et al*.^23^ demonstrated significantly better outcomes for intraosseous PRP administration compared to simple intra-articular injection (**Table 5**). Notably, these clinical improvements persisted through the final follow-up, 12 months in Sánchez *et al*.^53^ and 18 months in Su *et al*.^23^

Araujo *et al*,^51^ in their case series, evaluating intraosseous and IA-PRP injections, followed by 2 IA-HA injections, observed significant differences in both Western Ontario and McMaster Universities Osteoarthritis Index (WOMAC) and Visual Analog Scale (VAS) scores (*P* < 0.05) between the pre-operative and postoperative outcomes. The best outcomes were observed in younger age groups.

Lychagin *et al*.^52^ demonstrated that intraosseous PRP injections led to significant clinical improvement across all outcome measures at one year. Pain levels decreased notably on the VAS (*P* < 0.05), and knee function improved according to both the WOMAC and the Knee injury and Osteoarthritis Outcome Score (KOOS) scores. The most marked gains were seen in the KOOS subscales for sport/recreation and quality of life. Improvements were sustained over time, and MRI confirmed structural changes in BMLs.

Barman *et al*.^50^ showed how both groups, those receiving intra-articular PRP alone and those receiving combined intraosseous and intra-articular PRP, had significant improvements in pain and function over 6 months. Pain scores (VAS) and all KOOS subscales improved significantly within each group (*P* < 0.001). However, there were no significant differences between groups at 6 months, except for the sport/recreation subscale, which favored the IA-PRP group (*P* = 0.02)

Across the studies, a total of 5 minor adverse events (knee pain and swelling) were reported by Su *et al*,^23^ while Sánchez *et al*.^54^ noted only one case of knee swelling at 3 months post-treatment. Araujo *et al*.^51^ and Lychagin *et al*.^52^ did not report any adverse event in their papers. Barman *et al*^50^ reported that IO+IA-PRP consumed a greater amount of Acetaminophen.

Regarding conversion to TKA, Sánchez *et al*.^54^ reported that 2 of 14 patients presenting with grade III and IV KL OA (14.2%) progressed to joint replacement within 6 months. In another study^53^ by the same group, 5 patients (16.7%) in the intraosseous group required additional, though unspecified interventions, compared to 8 patients (26.7%) in the intra-articular group.

### Mesenchymal Stem Cells or Bone Marrow Derived Products

Our study identified 8 papers^42-49^ that investigated the subchondral injection of MSCs products (**Table 6**): 4 prospective trials,^42,47-49^ 3 RCTs^43-45^ and 1 retrospective study^46^

**Table 6.** MCSs.

Study	Follow-up (Months)	Results	Conversion to TKA	Complications	Need for Conservative Treatment
Vad *et al*.^42^	14	WOMAC +22.9, NRS +5.8, MRI: cartilage +14.1%	N/A	None	N/A
Hernigou *et al*.^45^	144	Similar to TKA; Cartilage +4.2%; BML regression	10% (3/30)	None in BMC group; TKA had more	None
Centeno *et al*.^49^	24	Improved pain and function (NPS, LEFS, and IKDC) in both groups; no significant difference between IO+IA versus IA	1 patient (IO+IA); 1 patient (IA)	No serious adverse events	12.5% (IO+IA) and 30% (IA) received additional PRP injections
Hernigou *et al*.^44^	180	Subchondral BMC more effective than IA for pain, MRI (BML regression, and cartilage volume ↑), function, and postponing TKA	20% (SC) versus 70% (IA); yearly incidence: 1.3% (SC) versus 4.6% (IA)	No serious adverse events	N/A
Hernigou *et al*.^43^	180	Subchondral BMC reduced pain and BML volume, improved cartilage; outcomes comparable to contralateral TKA	25/140 (18%) at mean 10 years; 1.19% per person-year	N/A	N/A
Pearl *et al*.^46^	24	BMAC chondroplasty in end-stage OA; only 1/23 knees requested TKA over 2 years; others had minor interventions	1/23 knees requested TKA (not yet performed)	N/A	5/23 required additional treatments (brace, steroids, viscosupplementation)
Kon *et al*.^47^	24	IKDC + 22.9. Similar improvements for all KOOS subscales. VAS +1.9; MRI↓bone marrow edema↑osteophytes, synovitis	3/30 patients (10%)	No major complications	1 patient had tibial osteotomy; others unspecified
Dallo *et al*.^48^	24	IKDC +35, KOOS +32, VAS +4; MRI complete or substantial resolution of BMLs	N/A	N/A	N/A

Vad *et al*.^42^ conducted a prospective study evaluating the efficacy of delivering 5 cm³ of tibial bone marrow concentrate (BMC) to the bone-cartilage interface of the affected knee using the PeCaBoo system under fluoroscopic guidance.

Centeno *et al*.^49^ compared intra-articular (IA) injection of bone marrow concentrate (BMC) and platelet products alone versus combined intraosseous plus intra-articular (IO+IA) injection for treating knee OA with bone marrow lesions (BMLs).

Hernigou et al. conducted 3 RCTs^43-45^ in BMC injections in patients with bilateral knee OA. In 2018,^45^ they compared BMC injection to TKA in the contralateral knee of patients with steroid-induced OA and osteonecrosis. In 2020,^44^ he compared IA versus subchondral BMC injections in opposite knees, using equal volumes from the same aspirate. Another 2021 study^43^ involved patients undergoing staged bilateral TKA, where one knee received TKA and the other a subchondral BMC injection, to assess whether baseline BMLs could predict progression to TKA.

Pearl *et al*.^46^ aimed to evaluate in their retrospective study whether bone marrow aspirate concentrate (BMAC) chondroplasty, where PRP obtained from blood and bone marrow aspirate from the ASIS were combined and injected under C-arm guidance into the femur and tibia, can delay the need for TKA in patients with advanced knee OA (KL 3-4), particularly in a veteran population.

In their prospective, multi-center trial Kon *et al*.^47^ involved 30 patients with unilateral symptomatic knee OA that received BMAC, injected both intra-articularly and into the subchondral bone (femoral condyle and tibial plateau) under fluoroscopic guidance to evaluate clinical and imaging outcomes.

On the other hand, Dallo *et al*.^48^ evaluated the clinical outcomes and safety of a minimally invasive biological subchondral bone augmentation procedure that combines core decompression, autologous bone graft, and bone marrow aspirate (BMA) injection, for the treatment of symptomatic bone marrow lesions (BMLs) in the knee.

## Clinical and MRI Findings

Regarding to the clinical outcomes, all studies reported improvement in patients’ reported scores (**Table 6**).

Vad *et al*.^42^ showed that significant differences in both WOMAC and Numeric Rating Scale (NRS) scores (*P* < 0.05) were observed in the follow-up time of 14 months after the treatment.

In terms of MRI outcomes, the author reported satisfactory restoration of the articular surface in 6 out of 10 patients with an average 23.5% increase of cartilage thickness.

Centeno *et al*.^49^ comparing the effectiveness of BMC and PRP of the 2 groups (IO + IA vs. IA) showed Numeric Pain Scale (NPS), Lower Extremity Functional Scale (LEFS) and International Knee Documentation Committee (IKDC) improved in both groups, but no statistical difference between them; both treatments were safe and well-tolerated; one patient per group eventually received knee arthroplasty. 12.5% (IO+IA) and 30% (IA) received additional PRP injections.

Hernigou *et al*.^45^ reported similar clinical outcomes between patients treated with the BMC and TKA. Most patients (21 out of 30) expressed greater satisfaction with the knee treated using BMC therapy. Another noteworthy observation was the significantly reduced MSC concentration in the osteonecrotic regions of the knee compared to the iliac crest, highlighting a biological deficiency within the subchondral osteonecrotic bone. At MRI findings, a mean increase in thickness of 4.2% was observed at their most recent follow-up (range 8-16 years). In addition, the authors documented a regression of BMLs in the femorotibial compartment of an average of 4.1 cm^3^ at 5 years’ follow-up, together with a significant decrease in the size of osteonecrosis by an average 40%. No adverse events in knees treated by intraosseous injections, whereas a much higher complication and reoperation rate was reported in the arthroplasty group, as expected for the type of procedure. Looking at the conversion rate, 3 knees that received BMC required TKA over the 12 year follow-up.

Hernigou *et al*.^44^ reported that subchondral (SC) injection was more effective in reducing pain, preserving cartilage, and regressing BMLs than IA injections. At 15 years, 20% of patients who underwent subchondral injection and 70% of patients who underwent IA injection had TKA, with an annual TKA rate of 1.3% SC versus 4.6% IA.

Patients preferred the SC-treated knee. Subchondral injection has been more successful in long-term postponement of TKA and joint preservation.

The same author in his prospective randomized study^43^ reported that at a 15-year follow-up, 18% of knees treated with subchondral MSC injections required TKA, with conversion occurring on average 10 years after treatment. The annual conversion rate (1.19% per person-year) was similar to the revision rate of the contralateral TKA knees. Cartilage volume increased by 2.3% at 2 years, though the new tissue was fibrocartilage. Despite similar function scores, more than half of the patients preferred the MSC-treated knee due to better pain relief.

Pearl *et al*,^46^ in their retrospective study, only 5 knees (21.7%) required any form of additional intervention within a 2-year follow-up period. Among these, one patient (4.3%) requested a TKA, although the surgery had not been performed by the time of the study’s conclusion. The other interventions included 2 steroid injections, one viscosupplement injection, and one use of an offloading brace.

Kon *et al*.^47^ reported how their procedure led to significant clinical improvements in knee OA patients, with IKDC and KOOS scores improving and remaining stable up to 24 months. VAS pain scores improved significantly at 12 months but showed some worsening by 24 months, though still better than baseline. MRI revealed a significant reduction in bone marrow edema, while other structural changes, including osteophyte growth and synovitis, worsened over time. Radiographs showed no OA progression in most cases. Ten percent of patients required TKA.

Dallo *et al*.^48^ showed how the use of their technique, combining core decompression, autologous bone graft and BMA injection, led to statistically significant improvements in clinical outcomes at 6 and 12 months postoperatively. Specifically, the IKDC score, KOOS, and VAS pain score all showed significant improvements from baseline (*P* < 0.05). No complications or adverse events were observed, indicating that the procedure is safe. Additionally, MRI follow-up at 12 months demonstrated resolution or substantial improvement in BMLs in all patients. Subgroup analyses revealed that improvements were observed regardless of age or body mass index. The treatment showed promise, particularly in patients with early to moderate OA (KL grade 2–3), suggesting it may help delay or prevent progression to TKA.

## Discussion

Intraosseous (IO) injections have gained increasing attention as a treatment option for knee OA, particularly with the use of bone substitutes (CaP) and biologic agents such as PRP and MSCs. The present systematic review investigated clinical outcomes, safety, and conversion rates to TKA following IO injections, with the aim of updating and expanding current knowledge on this evolving therapeutic strategy. The most comprehensive review prior to this work was conducted by Di Matteo *et al*.^8^, who analyzed 12 studies published up to January 2020, reporting encouraging short-term results while emphasizing the limited availability of high-quality evidence. The main finding of the present systematic review is that the use of IO injections for the symptomatic treatment of knee OA appears to be safe, feasible and clinically effective. However, these conclusion are tempered by the generally modest quality of the available evidence, with MSC products offering the most compelling long-term data. Notably, 5 randomized controlled trials (RCTs)^23,43-45,50^ were identified, representing a significant step forward in the field. However, limitations persist, including small sample sizes, heterogeneity in patient populations, particularly regarding the severity of OA (ranging from early to advanced stages), and generally short follow-up periods in most studies. These factors contributed to the modest average scores on the modified Coleman Methodology Score, and among the 5 RCTs included, only one^44^ out of the 5 was assessed as having a low overall risk of bias according to the RoB 2 tool. This finding underscores a general methodological concern among the available RCTs.

OA is the most common progressive musculoskeletal disorder, mainly affecting weight-bearing joints such as the hips and knees. Knee OA is increasingly recognized as a complex disorder that involves not just the deterioration of articular cartilage, but also pathological changes in multiple joint structures. This multifaceted involvement underscores the concept of knee OA as a condition that affects the entire joint organ rather than being limited to a single tissue.^56-62^

There is growing recognition that the subchondral bone plays a critical role in the initiation and progression of OA, as well as other degenerative and post-traumatic joint disorders. BMLs, visible on MRI, have been shown to correlate with pain, functional impairment, and accelerated structural joint damage.^63-65^

Intraosseous injections were therefore introduced with the aim of targeting pathological changes in the subchondral bone. The primary goal of subchondral treatment is not merely to delay joint replacement, but to restore the homeostasis and health of the osteochondral unit by modifying the joint’s biological environment.^49,64-66^

Two main strategies address knee subchondral bone pathology: CaP intraosseous injections for mechanical support and bone mineralization, and biologic treatments, using platelet-derived factors or MSCs to stimulate regeneration. While both aim to restore subchondral health, CaP provides immediate structural reinforcement, potentially reducing micromotion and pain, whereas biologics promote healing without enhancing short-term mechanical stability^39,55,49,67-69^

Currently, CaP has the most extensive representation in the literature (10 studies), compared to MSCs products and to PRP, which are addressed in 8 and 6 studies, respectively. Compared to 5 years ago, when Di Matteo *et al*.^8^ provided an initial overview of subchondral injectable therapies, there has been a notable increase in the number of randomized controlled trials (RCTs) and a broader adoption of biological products, particularly MSC products. However, no head-to-head comparisons have been conducted thus far. High-quality randomized trials are still needed to determine whether one of these substances offers superior or more durable outcomes. Additionally, there is no established consensus regarding the optimal volume of material to be injected into the affected subchondral bone, nor is it clear whether this dosage correlates with the extent of bone marrow lesions (BMLs) seen on MRI.

It is important to highlight the practical advantage that CaP bone substitutes are off-the-shelf products, readily available in the operating room, while autologous biologics require harvesting and immediate preparation, resulting in longer surgical times. Moreover, the use of MSCs is tightly regulated, prompting the adoption of “minimal manipulation” techniques to allow same-day processing in the operating room to be used on the patient.^70,71^

Technically, intraosseous knee injections are usually guided by fluoroscopy, which does not ensure accurate cannula placement within the BML, as lesion size and location can only be fully assessed via MRI. This limitation may lead to misplacement, especially in small lesions. To improve accuracy, MRI-based planning software and patient-specific targeting tools have been developed, although their use is not yet standard due to added cost.^72,73^

Arthroscopy is generally recommended when using CaP substitutes to detect possible IA leakage, which may cause inflammation. It also allows for concurrent procedures such as cartilage repair or meniscal treatment. However, in the context of biologics, arthroscopy is discouraged due to the risk of joint irritation and infection, and because fluid may dilute PRP or BMC, reducing their efficacy. Notably, 2 studies have shown that subchondral PRP injections lead to better outcomes than IA injections alone, highlighting the need to directly address subchondral bone damage.^34,74,75^

The use of CaP bone substitutes is widely used. This approach aims to restore subchondral bone stiffness and architecture through osteoconductive filler materials. While most studies reported meaningful improvements in pain and function, the outcomes were highly heterogeneous. Notably, Chatterjee *et al*.^35^ reported a high TKA conversion rate (45% at 12 months), suggesting limited benefit in certain patient populations or possibly suboptimal patient selection. On the other hand, studies such as Di Matteo *et al*.^41^ and Stratton *et al*.^39^ demonstrated low conversion rates (1.3% and 11%, respectively), highlighting the importance of technique standardization and strict inclusion criteria. Arthroscopic control was inconsistently employed, and the presence of concurrent IA procedures in some cases, as underlined by Randelli *et al*.^40^ in their study, further confounded outcome attribution. The overall safety profile of CaP intraosseous knee injection was favorable, with a low incidence of complications. Most adverse events were minor and self-limiting, although cases of IA cement leakage were occasionally reported. The relatively frequent need for retreatment with conservative IA therapies (e.g., HA or corticosteroids) in some cohorts raises questions about the long-term durability of SCP alone.

The subchondral delivery of BMC offers a unique regenerative strategy by providing MSCs, growth factors, and bioactive molecules directly into the site of subchondral dysfunction. Eight studies, including 3 RCTs by Hernigou *et al*,^43-45^ support the efficacy of this approach in improving clinical outcomes, regressing BMLs, and delaying joint replacement. The studies by Hernigou *et al*. are particularly notable for their long-term follow-up (up to 15 years) and rigorous design. These trials consistently demonstrated that subchondral BMC injections yielded superior structural and symptomatic outcomes compared to IA injections or even TKA on the contralateral limb in some cases. Annual TKA conversion rates as low as 1.19% were reported,^43^ substantially lower than historical controls for patients with BMLs. MRI evaluations in these studies confirmed meaningful reductions in lesion size and preservation of joint structure. At a 24-month follow-up, the conversion rate to arthroplasty reported by Kon *et al*,^47^ Pearl *et al*.,^46^ and Centeno *et al*.^49^ ranged between 2.5% and 10%. These relatively low rates of progression to TKA suggest that intraosseous injection therapies may serve as an effective strategy for delaying the need for joint replacement in appropriately selected patients. Across studies, BMC injections were safe and well-tolerated, with no severe adverse events directly attributable to the intraosseous technique. Although more complex and demanding in terms of preparation and application, bone marrow concentrate (BMC) therapy may be especially appropriate for younger, active patients with early-stage OA or post-traumatic subchondral lesions who seek to delay or avoid TKA.

Intraosseous PRP injection appears to offer promising results, particularly when compared to IA administration alone. Across 6 included studies, patients consistently reported improvements in pain and function, with some evidence of structural improvements in BMLs on MRI. Sánchez *et al*.^55^ and Su *et al*.^23^ demonstrated that IO-PRP may outperform IA-PRP in both symptom relief and durability of response. However, the heterogeneity in PRP preparation protocols, volume injected, and use of adjunctive IA injections limits direct comparability. Importantly, PRP therapy was associated with a low complication rate, with only mild transient symptoms such as swelling or discomfort reported. Conversion to TKA was reported in only a small subset of patients, though long-term data remain limited. These findings suggest that PRP injections into the subchondral bone may represent a safe and minimally invasive option for patients with early to moderate OA, especially those with MRI-detected BMLs.

Overall, the available evidence indicates that intraosseous (IO) treatments aimed at the subchondral bone can lead to improvements in pain and function for certain patients with knee OA and related conditions. Of the 3 approaches examined, bone marrow concentrate (BMC) seems to deliver the most consistent and durable results, though it involves a more technically demanding procedure. PRP offers a less invasive and safer option, while subchondroplasty with CaP substitute may help specific patient groups but shows more inconsistent outcomes and carries a higher risk of complications, when compared to the others. Notably, this is the most comprehensive analysis to date on knee intraosseous injections, and it demonstrates that these treatments are clinically effective, with consistent improvements across studies despite their overall modest methodological quality. From a clinical standpoint, knee intraosseous injections, irrespective of the specific substance used, appear to be safe and potentially effective in providing symptomatic relief and functional improvement in patients with OA, especially in the presence of bone marrow lesions (BMLs), and may be a valuable strategy to delay or avoid joint replacement in selected patient populations.

A major limitation of the current evidence base relates to the lack of standardized criteria for patient selection. Across studies, intraosseous injections were applied to heterogeneous OA populations, with some investigations requiring the presence of bone marrow lesions and others adopting broader indications without detailed MRI-based characterization. Lesion size, number, and anatomical location were rarely quantified using standardized methods, and the prerequisite failure of conservative treatment was inconsistently reported. This variability substantially limits the ability to define clear indications for intraosseous therapies and reduces the generalizability of the reported outcomes to routine clinical practice. Furthermore, interpretation of conversion to TKA should be approached with caution, as most studies did not report time-to-event analyses, such as hazard ratios or Kaplan–Meier estimates, nor provided sufficient data to calculate person-years. Consequently, conversion was generally reported as crude rates, reflecting current reporting practices but limiting meaningful comparisons across studies and highlighting the need for standardized survival analyses in future research.

An additional confounding factor is the frequent use of concomitant procedures, particularly in studies evaluating CaP intraosseous injections. Arthroscopy, cartilage repair techniques, meniscal procedures, and combined IA injections were commonly performed alongside subchondral treatments, making it difficult to isolate the independent effect of the intraosseous intervention. This issue is especially relevant when interpreting both clinical improvements and conversion rates to arthroplasty, as concurrent procedures may have contributed substantially to the observed outcomes. More broadly, the literature remains limited by substantial heterogeneity in injection protocols, outcome measures, and follow-up durations, as well as by the small number of high-quality RCTs and the scarcity of long-term follow-up beyond 2 years. Finally, the translation of subchondral therapies into routine clinical practice is hindered by high procedural and product costs and by the lack of dedicated cost-effectiveness analyses, particularly for biologic treatments, where product variability and limited standardization remain major concerns. Well-designed randomized trials with standardized indications, protocols, and extended follow-up are therefore required to clarify the long-term efficacy, cost-effectiveness, and clinical role of intraosseous therapies within joint-preserving treatment strategies.

## Conclusion

Intraosseous injections appear to be a safe, feasible, and potentially effective option for symptom relief in selected patients with knee OA, particularly in the presence of subchondral bone involvement. Among the available treatments, bone marrow concentrate demonstrates the most promising long-term results, while PRP and CaP injections may offer clinical benefits with a favorable safety profile. However, heterogeneity in patient selection, inconsistent characterization of BMLs, and the frequent use of concomitant procedures limit the ability to define clear treatment indications or to support broad clinical recommendations. High-quality RCTs with standardized inclusion criteria and long-term follow-up are needed to better clarify the role of intraosseous therapies in joint-preserving strategies.

## Appendix A. Complete Search Strategies

### PubMed (Searched February 12, 2025)

(“calcium phosphate” OR “tricalcium” OR “bone substitute” OR “bony substitue” OR “PRP” OR “ACP” OR “platelet rich” OR “platelet derived” OR “platelet concéntrate” OR “growth factor” OR “stem cells” OR “mesenchymal” OR “adipose” OR “BMC” OR “bone marrow concentrate” OR “SVF” OR “ AccuFill”) AND (“subchondroplasty” OR “subchondral injection” OR “subchondral” OR “intraosseous” OR “knee injection” OR “knee intraosseous injection”)

**Filters applied**: clinical trials, randomized controlled trials, controlled clinical trials, prospective studies, retrospective studies, cohort studies, case–control studies, comparative studies, and observational studies. Excluded: case reports, surveys or questionnaires, editorials, letters, comments, opinions, viewpoints, special topics, conference abstracts or proceedings, meeting abstracts, narrative reviews, systematic reviews, and meta-analyses.

**Date limits**: None

**Language restrictions**: English

### Embase (Searched February 19, 2025)

(“calcium phosphate” OR “tricalcium” OR “bone substitute” OR “bony substitue” OR “PRP” OR “ACP” OR “platelet rich” OR “platelet derived” OR “platelet concéntrate” OR “growth factor” OR “stem cells” OR “mesenchymal” OR “adipose” OR “BMC” OR “bone marrow concentrate” OR “SVF” OR “ AccuFill”) AND (“subchondroplasty” OR “subchondral injection” OR “subchondral” OR “intraosseous” OR “knee injection” OR “knee intraosseous injection”)

**Filters applied**: clinical trials, randomized controlled trials, controlled clinical trials, prospective studies, retrospective studies, cohort studies, case–control studies, comparative studies, and observational studies. Excluded: case reports, surveys or questionnaires, editorials, letters, comments, opinions, viewpoints, special topics, conference abstracts or proceedings, meeting abstracts, narrative reviews, systematic reviews, and meta-analyses.

**Date limits**: None

**Language restrictions**: English

### Cochrane Library (Searched February 25, 2025)

(“calcium phosphate” OR “tricalcium” OR “bone substitute” OR “bony substitue” OR “PRP” OR “ACP” OR “platelet rich” OR “platelet derived” OR “platelet concéntrate” OR “growth factor” OR “stem cells” OR “mesenchymal” OR “adipose” OR “BMC” OR “bone marrow concentrate” OR “SVF” OR “ AccuFill”) AND (“subchondroplasty” OR “subchondral injection” OR “subchondral” OR “intraosseous” OR “knee injection” OR “knee intraosseous injection”)

**Filters applied:** clinical trials, randomized controlled trials, controlled clinical trials, prospective studies, retrospective studies, cohort studies, case–control studies, comparative studies, and observational studies. Excluded: case reports, surveys or questionnaires, editorials, letters, comments, opinions, viewpoints, special topics, conference abstracts or proceedings, meeting abstracts, narrative reviews, systematic reviews, and meta-analyses.

**Date limits:** None

**Language restrictions:** English
